# Cutaneous Vasculitis and Digital Ischaemia Caused by Heterozygous Gain-of-Function Mutation in *C3*

**DOI:** 10.3389/fimmu.2018.02524

**Published:** 2018-11-01

**Authors:** Ebun Omoyinmi, Iman Mohamoud, Kimberly Gilmour, Paul A. Brogan, Despina Eleftheriou

**Affiliations:** ^1^Infection, Inflammation and Rheumatology Section, UCL Great Ormond Street Institute of Child Health, London, United Kingdom; ^2^Great Ormond Street Hospital NHS Foundation Trust, London, United Kingdom; ^3^Clinical Immunology Laboratory, Great Ormond Street Hospital NHS Foundation Trust, London, United Kingdom; ^4^Centre for Adolescent Rheumatology, Arthritis Research UK, University College London (UCL), University College London Hospital (UCLH) and Great Ormond Street Hospital (GOSH), London, United Kingdom

**Keywords:** cutaneous vasculitis, digital ischaemia, autoinflammation, gain-of-function (GOF), next-generating sequencing, targeted gene capture, complement component (C3, C4)

## Abstract

It is now increasingly recognized that some monogenic autoinflammatory diseases and immunodeficiencies cause vasculitis, although genetic causes of vasculitis are extremely rare. We describe a child of non-consanguineous parents who presented with cutaneous vasculitis, digital ischaemia and hypocomplementaemia. A heterozygous p.R1042G gain-of-function mutation (GOF) in the complement component C3 gene was identified as the cause, resulting in secondary C3 consumption and complete absence of alternative complement pathway activity, decreased classical complement activity, and low levels of serum C3 with normal C4 levels. The same heterozygous mutation and immunological defects were also identified in another symptomatic sibling and his father. C3 deficiency due GOF *C3* mutations is thus now added to the growing list of monogenic causes of vasculitis and should always be considered in vasculitis patients found to have persistently low levels of C3 with normal C4.

## Background

The complement system is an important component of the innate immune system, with several versatile functions in host defense, immune surveillance, and cell homeostasis (1–3). Deficiencies of individual complement proteins and complement regulatory proteins are associated with increased risk of infection, and in some cases autoimmunity (4, 5). C3 deficiency is very rare, with less than 50 cases described worldwide with varied clinical presentation (6–8). Primary C3 deficiency due to bi-allelic loss of function mutations in *C3* is typically associated with increased susceptibility to bacterial infections (such as bacterial pneumonia or meningitis) often manifesting in early childhood; and/or development of immune complex–mediated diseases such as glomerulonephritis (9–12). Additionally, polymorphisms in the *C3* gene may confer an increased risk for the development of age-related macular degeneration, atypical hemolytic uremic syndrome (aHUS), dense deposit glomerulonephritis (13–16), or influence outcomes of organ transplantation (17, 18). Whilst reduced plasma C3 is commonly observed with concomitant reduction in C4 due to classical pathway activation, for example secondary to autoimmune diseases such as systemic lupus erythematosus, low plasma C3 with relative conservation of C4 is typical of alternative pathway activation, and should always prompt further investigation for monogenic causes (19, 20). These include bi-allelic loss of function mutations in C3, or complement factor I (*CFI*) (19), but can also be caused by mono allelic gain-of-function (GOF) *C3* mutation (13, 21, 22). Vasculitis caused by GOF mutation in *C3* has never been reported before, however. Herein, we describe a child of non-consanguineous parents who presented with cutaneous vasculitis, digital ischaemia and hypocomplementaemia. A heterozygous p.R1042G GOF mutation of *C3* was identified as the cause, resulting in complete absence of alternative complement pathway activity, reduced classical complement activity, and low levels of C3 with normal C4 levels.

## Case presentation

**Figure 1:**
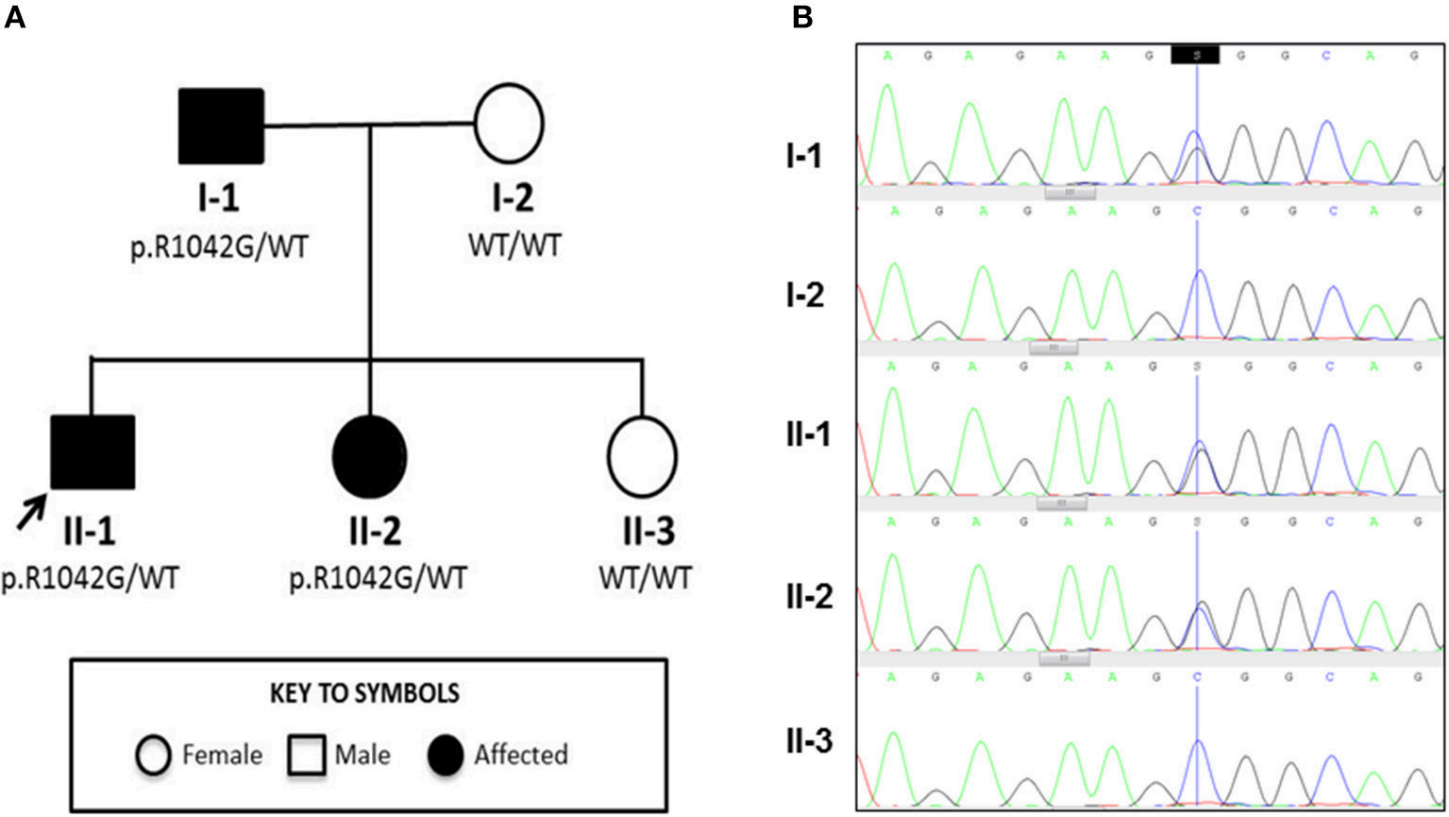
Family tree and Sanger sequencing results**. (A)** Family tree showing affected and unaffected members enrolled in the study; segregation of the p.R1042G mutation in *C3* with disease is also indicated. Arrow indicates the index case. **(B)** Sanger sequencing confirmed a heterozygous C/G mutation (blue/black overlapping line) at position c.C3124 of *C3* gene in family members II-1, I-1, and II-2 that is absent (single blue line corresponding to wild type “C” allele) in I-2 and II-3.

**Figure 2:**
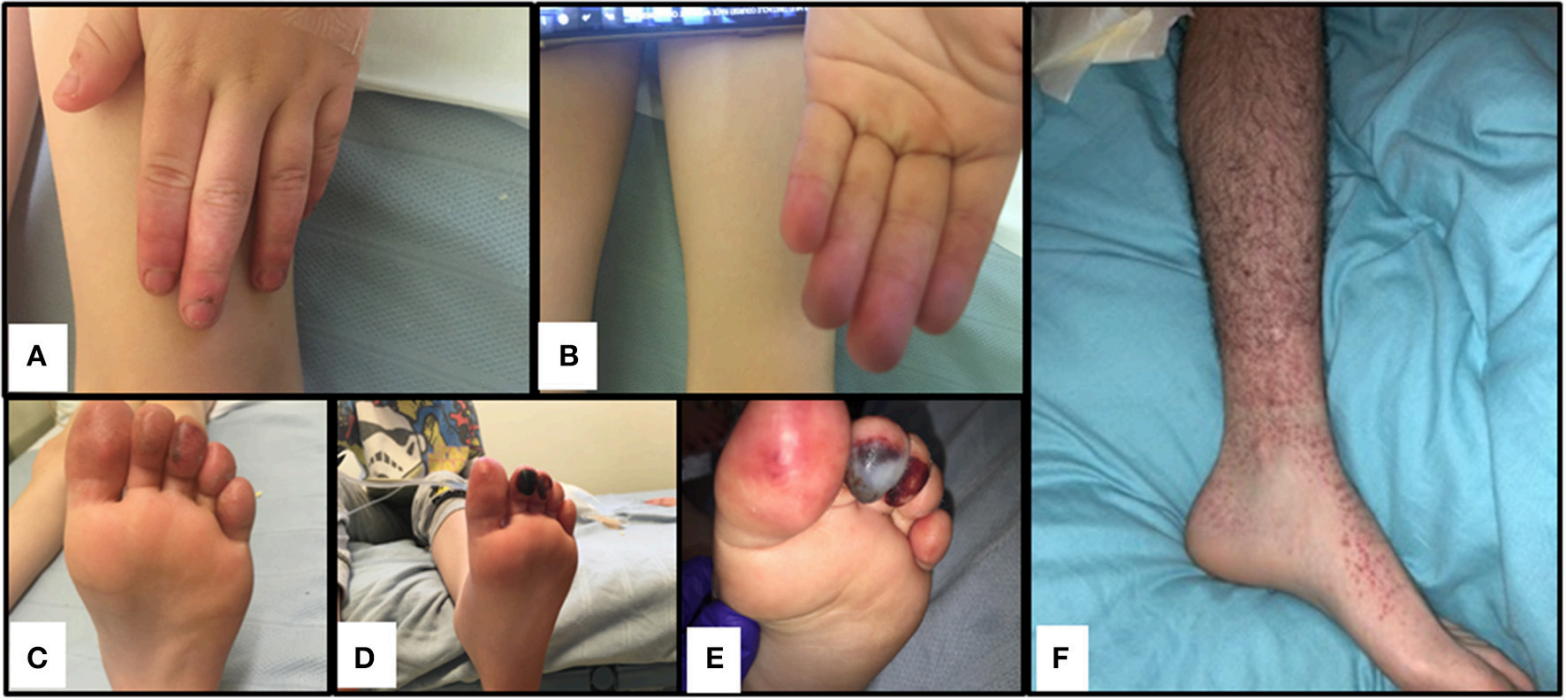
Cutaneous vasculitis and digital ischaemia associated with heterozygous gain-of-function mutation in C3. **(A–C)**. Discolouration and erythema in fingers of both hands and toes of the index case (II-1). **(D,E)**. Digital ischaemic necrosis seen in the second and third digit of the left foot for II-1. **(F)** Cutaneous vasculitis affecting I-1.

The index case was a previously well 6-year-old male, born to non-consanguineous Caucasian parents (family tree, Figure 1A). He spontaneously developed acute, painful erythema and discoloration of his fingers and toes (Figures 2A–C). There were no reported precipitants, specifically no evidence of any intercurrent infection, and no past medical history suggestive of immunodeficiency. On systems review, he reported intermittent non-peritonitic abdominal pain, and arthralgia of knees and ankles. He rapidly deteriorated over the next 48 h, developing critical digital ischaemia of his toes (Figures 2D,E).

Laboratory investigations (Supplemental Table 1) in the proband revealed normal renal function and blood pressure, and there was no evidence of proteinuria or other organ specific involvement. Chest radiograph, abdominal ultrasonography, echocardiography, and visceral digital subtraction catheter arteriography were all normal. There was only minor elevation of the erythrocyte sedimentation rate (13 mm/hour; reference range [RR] 0-10), and normal C-reactive protein (CRP) < 5 mg/L (RR < 20). All full blood count parameters were normal. Blood film examination was unremarkable. He had low titer antinuclear antibodies (1:160). Other autoantibodies (rheumatoid factor, ANCA, including anti-proteinase 3 and anti-myeloperoxidase; anti-double stranded DNA; anticardiolipin antibodies and lupus anticoagulant; thyroid peroxidase antibodies; and antibodies against extractable nuclear antigens) were all negative. Extensive investigations for an infectious cause of his symptoms were negative, specifically negative mycoplasma pneumoniae serology; and he had negative cryoglobulins. A full prothrombotic workup was also negative. Notably, however, he had persistently low serum C3 (0.22 g/L; RR 0.75–1.65), and normal C4 (0.21 g/L; RR 0.14–0.54); complete absence of alternative complement functional activity (0%, RR >10%); and reduced functional classical pathway activity (31%; RR> 40%; Table 1). Low C3, normal C4, absent alternative complement pathway activity, and markedly decreased classical complement pathway activity persisted, and prompted more detailed scrutiny of the complement pathway (see below for complement deficiency work up). Vasodilatory treatment for critical digital ischaemia included oral nifedipine 40 mg/day with little response, and subsequently prostacyclin infusion (20 ng/mg/min continuous infusion over 5 days) to improve the perfusion in his toes. He was also treated with a short course of oral prednisolone (2 mg/kg/day; weaning over 12 weeks), aspirin (5mg/kg /day) and oral azathioprine (2 mg/kg/day), on which he currently remains 12 months later. He made a full recovery with no residual skin lesions or permanent tissue loss. Corticosteroids however were reintroduced with good response 13 months later as there was recurrence of finger ischaemia. C3 remains persistently decreased 0.22 g/L, with normal C4 0.27 g/L despite ongoing treatment.

**Table 1:** Complement assays in the index case and other family members.

**Case**	***C3* Genotype**	**CFI mg/L (%healthy control)**	**CFH mg/L (%healthy control)**	**C1q mg/L (RR 50-250)**	**C1q autoAb U/ml (RR 0-15)**	**C3 g/L (RR 0.75–1.65)**	**C4 g/L (RR 0.14–0.54)**	**Classical complement pathway assay (RR >40%)**	**Alternative complement pathway assay (RR >10%)**	**MBL ng/ml (RR >1300)**
Index case (II-1)	Heterozygous p.R1042G	86	131	126	>400	0.22	0.21	31%	0%	> 4000
Sister(II-2)	Heterozygous p.R1042G	84	148	115	25	<0.23	0.27	0%	0%	3138
Sister(II-3)	Wild type	140	97	164	6	1.53	0.27	44	10	>4000
Father(I-1)	Heterozygous p.R1042G	109	161	62	>400	<0.23	0.28	31	0	3120
Mother(I-2)	Wild type	77	146	141	<0.6	1.28	0.23	45	25	3388

*Abnormal results shaded gray. CFH, complement factor H; CFI, complement factor I; RR, reference range; U/ml, units per milliliter; AutoAb, autoantibody; MBL, mannose binding lectin*.

### Work up for suspected complement deficiency

Table 1 summarizes the genotype and results of complement studies. All experimental work was performed with ethical approval (ethics number: 08H071382) and with written informed consent from all adult participants, assent (where appropriate) for children, and parental consent for children. C3 and C4 measurements were performed using a standard nephelometry assay (BN II Siemens Healthcare UK). Classical and alternative complement assays (EuroDiagnostics, Sweden); MBL assays (BioPorto) were performed by enzyme-linked immunosorbent assay (ELISA). Complement factor I and H were measured at Pathology Imperial College Healthcare, NHS, UK using ELISA (Binding Site, UK). C1q was measured by radial immunodiffusion (RID) and C1q antibodies by ELISA at the Protein Reference Unit Sheffield, UK.

Genetic investigation of the index case (II-1) was performed using our recently developed targeted gene panel for Vasculitis and AutoInflammation Panel (VIP) that contains 201 genes, including 19 pertaining to complement and regulatory proteins (Supplemental Table 2) (23). This revealed a heterozygous c.3124C>G (p.R1042G) variant in exon 24 of *C3* gene, which was confirmed by Sanger sequencing (Figure 1B). This variant is not reported in the population databases (1000 genomes, ESP 6500, and ExAC) and is predicted to be damaging according to 3 different *in silico* analysis; SIFT [D], PolyPhen2 [D], and MutationTaster [D]. There were no other class 4 or 5 genetic variants detected in any of the other genes included in the targeted panel for the index case.

Detailed complement studies were undertaken in other first-degree family members and showed low C3 levels, absent alternative complement pathway activity, and markedly decreased classical complement pathway activity in II-2 and I-1. These studies were normal for I-2 and II-3 (Table 1). Sanger sequencing in all enrolled family members confirmed that the heterozygous p.R1042G *C3* mutation was also present in both II-2 and I-1 and absent in I-2 and II-3 (Figures 1A,B). Interestingly I-1 subsequently developed recurrent cutaneous vasculitis (purpuric rashes) and arthralgia (Figure 2F) with no documented renal abnormalities and required corticosteroid therapy. II-2 was also reporting long standing history of recurrent fingertip erythema with no other systemic symptoms while I-2 and II-3 were asymptomatic (see Supplemental Table 2 for other laboratory tests for I-1 and II-2).

## Discussion

We describe a monoallelic p.R1042G mutation in *C3* as the genetic cause of familial cutaneous vasculitis and severe digital ischaemia. This mutation has been previously described as a GOF mutation, associated with glomerulonephritis (24). The presentation in our patient with digital vasculitis and ischaemia is therefore unique, and the vasculitic features in other family members who had the same mutation and immunophenotype emphasizes that this is a fully penetrant dominant mutation in this kindred. Thus the spectrum of monogenic vasculitis continues to expand, and mono-allelic *C3* GOF mutations should now be considered in patients with ANCA negative vasculitis, particularly when associated with low serum C3, but normal C4 levels.

**Figure 3:**
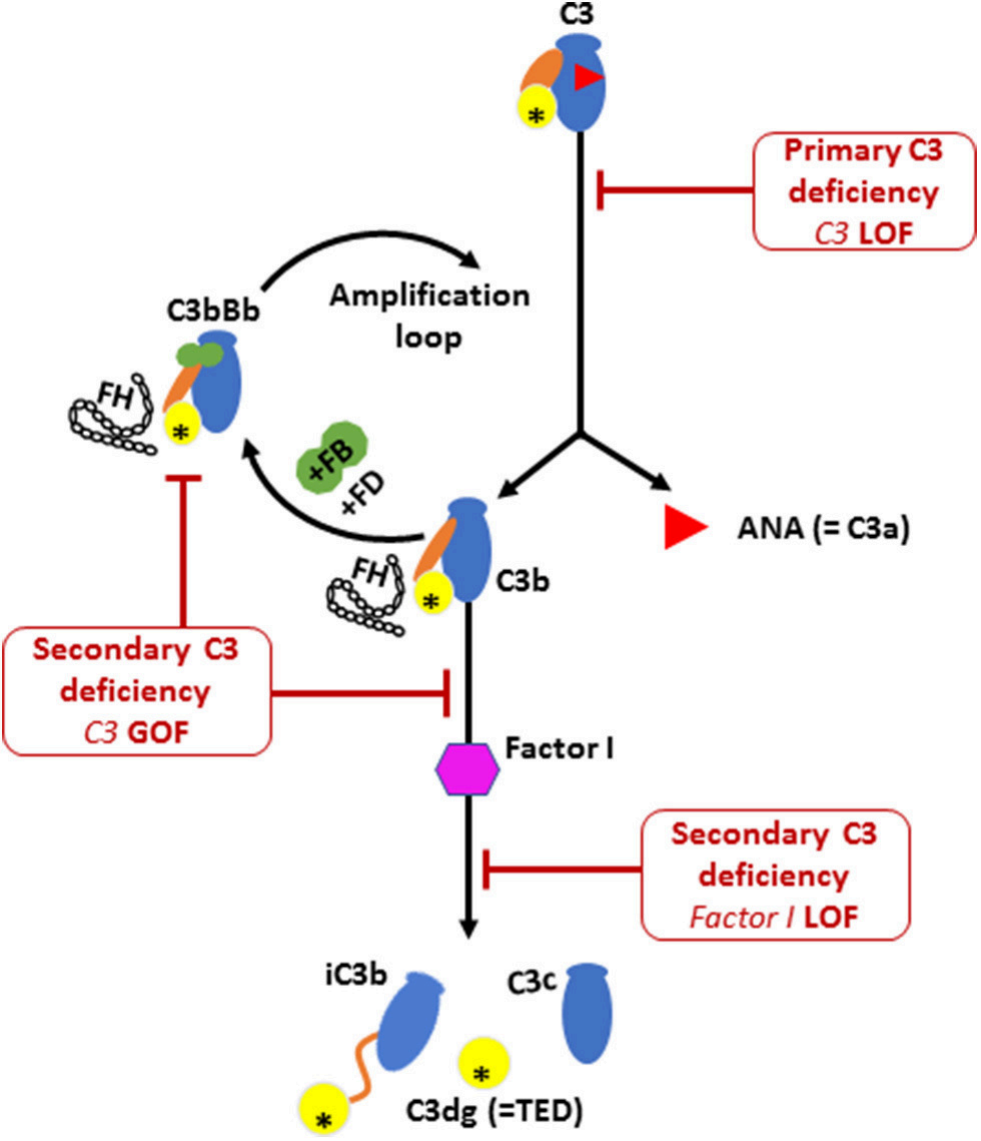
The activation and regulation of the alternative pathway (AP) complement component C3. Illustration of C3 molecule is presented in blue, with ANA domain (C3a) in red triangle, yellow circle for TED domain, and orange oval for the CUB domain. Major structural changes occurs during C3 activation leading to the release of bioactive fragments C3a and C3b. Alternative pathway C3 convertase (C3bBb) is then formed by the association of C3b fragment with complement factor B (FB; illustrated in green) in the presence of complement factor D (FD). Further activation of C3 then occurs via the amplification loop. The consumption of AP C3 is regulated by Factor I that degrades C3b and Factor H that dissociate C3 convertase. Factor H is also required for the protease activity of Factor I. Low plasma C3 is associated with bi-allelic loss of function (LOF) mutations in C3 or complement factor I (CFI) and also by mono allelic gain-of-function (GOF) C3 mutation. The C3 p.R1042G mutation (indicated by an asterisk in the TED domain) leads to the formation of an abnormal C3 convertase that cannot bind to FH, thereby amplifying the consumption of C3.

During the activation process of the alternative complement pathway, C3 undergoes large conformational changes following the release of C3a by C3 convertase, thereby exposing the reactive thio-ester containing domain (TED) on the C3b fragment (Illustrated in Figure 3). The TED on C3b can then rapidly covalently bind to nucleophiles on cell surfaces (25). Attachment of the C3b fragment to cell surfaces initiates a powerful amplification reaction, by interaction with factor B, factor D and properdin, leading to the formation of more C3 convertases that use plasma C3 as a substrate (26, 27). This positive feedback amplification loop, if dysregulated, results in rapid and uncontrolled consumption of C3, with major impact on the alternative complement pathway (28, 29), and relative sparing of the classic pathway which can function with much lower levels of C3 than the alternative pathway (19). The heterozygous p.R1042G *C3* mutation in our patients is predicted to affect the active TED in the C3 protein. Structural studies have demonstrated that this mutation is positioned within the interacting interface of C3 TED domain with Factor H consecutive complement control protein (CCP) domains, thereby inhibiting the regulatory binding of FH to the alternative pathway C3 convertase (i.e., C3bBb) to suppress the amplification loop (30, 31). Thus, the p.R1042G *C3* mutation is predicted to reduce plasma C3 levels by generating abnormal alternative pathway C3 convertases (C3bBb) which cannot be inactivated by FH, and leading to rapid consumption of the normal C3 encoded by the normal allele (24, 30).

The heterozygous p.R1042G *C3* mutation we identified in our kindred was previously described in a single case of a 45 year old Spanish male patient who developed mild proteinuria and microscopic haematuria, but who had no other symptoms or cutaneous manifestations (24). At the time of writing, none of our patients have yet developed renal involvement, but close monitoring of blood pressure, renal function and urinalysis has been initiated in all family members with the heterozygous p.R1042G *C3* genotype. Whether renal histopathological abnormalities precede the development of more typical renal function abnormalities could not be established for our cases since a renal biopsy was not clinically indicated or justifiable. Of note, II-2, also heterozygote for the p.R1042G GOF *C3* mutation, has abnormal alternative and classical complement function, and low C3 levels but is currently presenting with a milder phenotype suggesting some variability in the type of clinical manifestations associated with GOF *C3* mutation. Additional genetic and/or environmental risk factors (such as intercurrent infection or other trigger) may play a role in modifying the observed phenotype. It is likely that complement regulatory molecules act as a protein network and that multiple hits, probably involving plasma and membrane associated complement regulatory proteins, are required to significantly impair protection to host tissues. As such an individual's complotype could influence susceptibility to, or severity of, diseases such as C3 deficiency (31–34).

Therapeutic options for the inflammatory manifestations associated with GOF *C3* mutations and resultant secondary C3 deficiency are very limited, but so far our index case has remained stable on treatment with vasodilation, anti-aggregation (aspirin) and immunosuppression. Since the effects of complement are associated with inflammatory sequelae (such as vasculitis) but also possible immune deficiency, we chose a medium strength immunosuppressant to ultimately work alongside corticosteroids eventually working as steroid sparing agent. More targeted complement-modulating agents such as eculizumab, or avacopan might be worthy of consideration, albeit with a limited scientific rationale/evidence-base. Eculizumab is a humanized monoclonal antibody that binds C5 and prevents assembly of the membrane attack complex (C5b-9) thereby blocking the final complement cascade and reducing uncontrolled activation of the alternative complement pathway (35). Several case reports and open-label studies have already shown a potential benefit of eculizumab for patients with C3 glomerulonephritis and aHUS where there is an acquired dysregulation of complement factors (35–38). In addition, avacopan, a C5a receptor inhibitor, downregulates activation of the alternative complement pathway and has been shown to be an effective add-on therapy for ANCA associated vasculitis (39, 40). Therefore, these complement modulating agents may also have a role in treating autoimmunity/autoinflammation associated with GOF *C3* mutations via reduction of uncontrolled activation of the alternate complement pathway. An alternative approach would be to use plasma exchange to remove dysfunctional C3. However, membrane-bound abnormal C3, which is responsible for alternative pathway activation, cannot be eliminated by plasma exchange, and continued supplementation of C3 may enhance further complement activation. Lastly, for other cases of C3 deficiency secondary to genetic defects affecting the complement regulatory proteins factor H and I, orthotopic liver transplantation has also been trialed albeit with variable success, given that these proteins are mainly produced in the liver. Such an approach, however, is unlikely to be relevant for patients with GOF *C3* mutations given that C3 is not only synthesized in the liver but is also synthesized by immune cells (41), adipocytes (42), and neuronal cells (43).

We felt that our patients suffered cutaneous vasculitis despite the lack of confirmatory biopsy findings. It is now well accepted that histological confirmation is not needed to diagnose vasculitis (44), and that clinical diagnoses based on a sound understanding of pathogenesis (in this case consumptive C3 disease from C3 gain of function and presumed immune complex formation injury to small vessels in the peripheries) provides justification to describe this as a vasculitis. In addition, the father (I-1) developed palpable purpura (see Figure 2F) and typical vasculitic lesions and has an ESR of 65 mm/h (see Supplemental Table 1); the sibling II-2 with more minor symptoms of the fingers also has an elevated ESR of 60 mm/h, confirming the inflammatory component to this pathology.

Lastly, we cannot completely exclude the possibility that anti-C1q antibodies might play some secondary role in (II-1 and I-1) to explain the observed phenotype, although their presence may represent a non-specific epiphenomenon secondary to chronically increased complement activation, particularly in view of normal C1q levels observed in these individuals, and the fact that C1q antibodies have also been observed non-specifically in complement factor deficiency (19).

In summary, we expand the spectrum of monogenic complement deficiencies that cause vasculitis by describing a family with monoallelic p.R1042G mutation in *C3* causing cutaneous vasculitis and digital ischaemia. Heterozygous GOF mutations in C3 are now added to the list of genetic complement deficiencies causing vasculitis such as C1q, C1r, and C1s deficiency, complement factor I deficiency and complement C2 and C4 deficiency (1, 7, 19). The therapeutic role of complement inhibition therapies to prevent or reduce tissue damage caused by dysregulated complement activation in this setting remains to be established.

## Ethics statement

Written informed consent was obtained from the participants for the publication of this report.

## Author contributions

EO, PB, and DE: design of the work, acquisition and analysis of data, drafting and revising manuscript, providing approval for publication; IM: acquisition and analysis of data, providing approval for publication; KG: acquisition and analysis of data, revising manuscript, providing approval for publication.

### Conflict of interest statement

The authors declare that the research was conducted in the absence of any commercial or financial relationships that could be construed as a potential conflict of interest.
