# Supplementary material for: Cutaneous Vasculitis and Digital Ischaemia Caused by Heterozygous Gain-of-Function Mutation in C3
Source: Front Immunol. 2018 Nov 1;9:2524. doi: 10.3389/fimmu.2018.02524 (PMC6221951; doi:10.3389/fimmu.2018.02524)
Supplement: Supplementary file 1 [file Table_1.DOCX]

**Table S1: Laboratory investigations summary for all affected patients.**

Summary of immunology, biochemistry and haematology investigations in the index case (II-1) and I-1, II-2.

| **Laboratory investigations** | **Patient II-1**  **(Reference range)** | **Patient I-1**  **(Reference range)** | **Patient II-2**  **(Reference range)** |
| --- | --- | --- | --- |
| Autoantibodies | ANA 1:160  Anti-C1q antibodies >400 U/ml (0-15 U/ml) | ANA 1:160  Anti-C1q antibodies >400 U/ml (0-15 U/ml) | Anti-C1q antibodies 25 U/ml (0-15 U/ml) |
| Haemoglobin | 114 g/L (120-160 g/L) | 139 g/L (120-160 g/L) | 98 g/L (120-160 g/L) |
| Platelet count | 306 x 10^9^/L (150-450 x 10^9^/L) | 368 x 10^9^/L (150-450 x 10^9^/L) | 307 x 10^9^/L (150-450 x 10^9^/L) |
| White blood cell count | 10.15 x 10^9^/L (4.0-11 x 10^9^/L) | 14.66 x 10^9^/L (4.0-11 x 10^9^/L) | 9.8 x 10^9^/L (4.0-11 x 10^9^/L) |
| Lymphocyte count | 3.30 x 10^9^/L (2.0-9.5 x 10^9^/L) | 2.81 x 10^9^/L (2.0-9.5 x 10^9^/L) | 3.15 x 10^9^/L (2.0-9.5 x 10^9^/L) |
| Neutrophil count | 4.69 x 10^9^ /L (1.8-8.0 x 10^9^/L) | 9.97 x 10^9^/L (1.8-8.0 x 10^9^/L) | 3.87 x 10^9^/L (1.8-8.0 x 10^9^/L) |
| Monocyte count | 0.77 x 10^9^ /L (0.1-0.8 x 10^9^/L) | 1.42 x 10^9^ /L (0.1-0.8 x 10^9^/L | 0.77 x 10^9^ /L (0.1-0.8 x 10^9^/L) |
| Immunoglobulin G | 15 g/L (4.9-16.1 g/L) | 13.10 g/L (4.9-16.1g/L) | 13.60 g/L (4.9-16.1 g/L) |
| Immunoglobulin A | 1.40 g/L (0.4-2 g/L) | 3.58 g/L (0.4-2 g/L) | 1.33 g/L (0.4-2 g/L) |
| Immunoglobulin M | 1.16 g/L (0.5-2.0 g/L) | 0.84 g/L (0.5-2.0 g/L) | 0.76 g/L (0.5-2.0 g/L) |
| Erythrocyte sedimentation rate | 13 mm/hr (<10 mm/hr) | 65 mm/hr (<10 mm/hr) | 60 mm/hr (<10 mm/hr) |
| Serum amyloid A | 3.2 mg/L (<10 mg/L) | 6.5 mg/L (<10 mg/L) | 3 mg/L (<10 mg/L) |
| C-reactive protein | <5 mg/L (<20 mg/L) | 6 mg/L (<20 mg/L) | 5 mg/L (<20 mg/L) |
| Urea | 5.7 mmol/L (2.5-6.0) mmol/L | 4.5 mmol/L (2.5-6.0) mmol/L | 5.3 mmol/L (2.5-6.0) mmol/L |
| Creatinine | 36 umol/L (25-42) umol/L | 81 umol/L (25-78) umol/L | 39 umol/L (25-42) umol/L |
