# Supplementary material for: Cutaneous Vasculitis and Digital Ischaemia Caused by Heterozygous Gain-of-Function Mutation in C3
Source: Front Immunol. 2018 Nov 1;9:2524. doi: 10.3389/fimmu.2018.02524 (PMC6221951; doi:10.3389/fimmu.2018.02524)
Supplement: Supplementary file 2 [file Table_2.DOCX]

**Table S2. List of genes included in Vasculitis and Autoinflammation gene panel.**

| **Gene symbol** | **Gene name** | **Transcript** |
| --- | --- | --- |
| ACP5 | Acid phosphatase-5/tartrate-resistant phosphatase | NM_001111034 |
| ACTA2 | Actin alpha 2 | NM_001613 |
| ADA2 | Cat eye syndrome chromosome region 1/Adenosine deaminase 2 | NM_001282225 |
| ADAM17 | ADAM Metallopeptidase Domain 17 | NM_003183 |
| ADAR | Adenosine deaminase acting on RNA | NM_001111 |
| AICDA | Activation-induced cytidine deaminase | NM_020661 |
| AIRE | Autoimmune Regulator | NM_000383 |
| AP1S3 | Adaptor Related Protein Complex 1 Sigma 3 Subunit | NM_001039569 |
| AP3B1 | Adaptor Related Protein Complex 3 Beta 1 Subunit | NM_003664 |
| APOA1 | Apolipoprotein A1 | NM_000039 |
| APOA2 | Apolipoprotein A2 | NM_001643 |
| APOA4 | Apolipoprotein A4 | NM_000482 |
| APOC2 | Apolipoprotein C2 | NM_000483 |
| APOC3 | Apolipoprotein C3 | NM_000040 |
| APOE | Apolipoprotein E | NM_000041 |
| APP | Amyloid Beta Precursor Protein | NM_000484 |
| B2M | Beta-2-Microglobulin | NM_004048 |
| BLOC1S6 | Biogenesis Of Lysosomal Organelles Complex 1 Subunit 6 (BLOC1S6) | NM_012388 |
| BMPR2 | Bone morphogenetic protein type II receptor | NM_001204 |
| BTK | Bruton's tyrosine kinase | NM_000061 |
| C1QA | Complement C1q A Chain | NM_015991 |
| C1QB | Complement C1q B Chain | NM_000491 |
| C1QC | Complement C1q C Chain | NM_172369 |
| C1R | Complement C1r | NM_001733 |
| C2 | Complement C2 | NM_000063 |
| C3 | Complement C3 | NM_000064 |
| C5 | Complement C5 | NM_001735 |
| C6 | Complement C6 | NM_000065 |
| C7 | Complement C7 | NM_000587 |
| C8A | Complement C8 Alpha Chain | NM_000562 |
| C8B | Complement C8 Beta Chain | NM_000066 |
| C9 | Complement C9 | NM_001737 |
| CARD14 | Caspase Recruitment Domain Family Member 14 | NM_024110 |
| CASP10 | Caspase 10 | NM_032977 |
| CASP8 | Caspase 8 | NM_033355 |
| CBL | Cbl Proto-Oncogene, E3 Ubiquitin Protein Ligase | NM_005188 |
| CBS | Cystathionine beta synthase | NM_000071 |
| CD40LG | CD40 antigen ligand | NM_000074 |
| CD70 | Tumor Necrosis Factor Ligand Superfamily Member 7 | NM_001252 |
| CFH | Complement factor H | NM_000186 |
| CFHR5 | Complement factor H-related protein 5 | NM_030787 |
| CFI | Complement factor 1 | NM_000204 |
| CFP | Complement Factor Properdin | NM_002621 |
| COL3A1 | Collagen Type III Alpha 1 Chain | NM_000090 |
| COL4A1 | Collagen Type IV Alpha 1 Chain | NM_001845 |
| COL5A1 | Collagen Type V Alpha 1 Chain | NM_000093 |
| COL5A2 | Collagen Type V Alpha 2 Chain | NM_000393 |
| COL7A1 | Collagen Type VII Alpha 1 Chain | NM_000094 |
| COPA | Coatomer subunit alpha | NM_001098398  NM_004371 |
| CORO1A | Coronin, actin binding protein, 1A | NM_007074 |
| CPT2 | Carnitine palmitoyltransferase 2 | NM_000098 |
| CST3 | Cystatin C3 | NM_000099 |
| CTC1 | CTS telomere maintenance complex component 1 | NM_025099 |
| CTPS1 | Cytidine 5′ triphosphate synthase 1 | NM_001905 |
| CYBA | Cytochrome b alpha chain | NM_000101 |
| CYBB | Cytochrome b beta chainp91-phox | NM_000397 |
| DCLRE1C | DNA cross-link repair 1c | NM_001033855 |
| DNASE1 | Deoxyribonuclease 1 | NM_005223 |
| DNASE1L3 | Deoxyribonuclease I-like 3 | NM_004944 |
| DNASE2 | deoxyribonuclease II, lysosomal | NM_001375 |
| DOCK8 | Dedicator of cytokinesis 8 | NM_203447 |
| EFEMP2 | EGF-containing fibulin-like extracellular matrix protein 2; also referred to as Fibulin 4; FBLN4 | NM_016938 |
| ELANE | Elastase, neutrophil-expressed | NM_001972 |
| ELN | Elastin | NM_001278939 |
| FAS | Tumour necrosis factor receptor superfamily member 6 | NM_000043 |
| FASLG | Tumor necrosis factor ligand superfamily member 6 (FAS ligand) | NM_000639 |
| FBN1 | Fibrillin 1 | NM_000138 |
| FBN2 | Fibrillin 2 | NM_001999 |
| FERMT1 | Ferritin family member 1 | NM_017671 |
| FGA | Fibrinogen Alpha Chain | NM_000508 |
| FLNA | Filamin A | NM_001456 |
| FOXE3 | Forkhead Box E3 | NM_012186 |
| FOXP3 | Forkhead box P3 | NM_014009 |
| G6PC3 | Glucose-6-phosphatase 3 | NM_138387 |
| GATA2 | GATA-binding protein 2 | NM_032638 NM_001145661 |
| GLA | Alpha-galactosidase A | NM_000169 |
| GSN | Gelsolin | NM_001127662 |
| GUCY1A3 | Guanylate Cyclase 1 Soluble Subunit Alpha | NM_000856 |
| GUCY2C | Guanylate cyclase 2C | NM_004963 |
| HFE | hemochromatosis | NM_000410 |
| HPS1 | Hermansky-Pudlak syndrome type 1 | NM_000195 |
| HPS4 | Hermansky-Pudlak syndrome type 4 | NM_022081 |
| HPS6 | Hermansky-Pudlak syndrome type 6 | NM_024747 |
| HTR1A | 5-Hydroxytryptamine Receptor 1A | NM_000524 |
| HTRA1 | HtrA serine peptidase-1 gene | NM_002775 |
| ICOS | Inducible T-cell co-stimulator | NM_012092 |
| IFIH1 | Interferon-induced helicase C domain-containing protein 1 | NM_022168 |
| IFNGR1 | Interferon gamma receptor 1 | NM_000416 |
| IFNGR2 | Interferon gamma receptor 2 (interferon gamma transducer 1) | NM_005534 |
| IKBKG | IKK-gamma | NM_003639 |
| IL10 | Interleukin 10 | NM_000572 |
| IL10RA | Interleukin 10 receptor, alpha | NM_001558 |
| IL10RB | Interleukin 10 receptor, beta | NM_000628 |
| IL1RN | Interleukin 1 receptor antagonist | NM_173842 |
| IL2RA | Interleukin 2 receptor, alpha chain | NM_000417 |
| IL31RA | Interleukin 31 Receptor A | NM_139017 |
| IL36RN | Interleukin 36 receptor antagonist | NM_173170 |
| IRF8 | Interferon Regulatory Factor 8 | NM_002163 |
| ISG15 | ISG15 Ubiquitin-Like Modifier | NM_005101 |
| ITGB2 | Beta-2 integrin chain | NM_000211 |
| LACC1 | Laccase domain containing 1 | NM_001128303 |
| LMNA | Laminin A | NM_170707 |
| LOX | Lysyl Oxidase | NM_001178102  NM_001317073  NM_002317 |
| LPIN2 | Lipin 2 | NM_014646 |
| LRBA | Lipopolysaccharide-responsive and beige-like anchor brotein | NM_001199282 |
| LYN | Tyrosine-Protein Kinase | NM_002350 |
| LYST | Lysosomal trafficking regulator | NM_000081 |
| LYZ | Lysozyme | NM_000239 |
| MAGT1 | Magnesium Transporter 1 | NM_032121 |
| MASP2 | Mannose-binding lectin serine protease 2 | NM_006610 |
| MAT2A | Methionine Adenosyltransferase 2A | NM_005911 |
| MBL2 | Mannose-binding lectin | NM_000242 |
| MEFV | MEditerranean FeVer | NM_000243 |
| MFAP5 | Microfibrillar Associated Protein 5 | NM_003480 |
| MVK | Mevalonate Kinase | NM_000431 |
| MYD88 | Myeloid Differentiation Primary Response 88 | NM_001172569  NM_002468 |
| MYH11 | Myosin, Heavy Chain 11, Smooth Muscle | NM_001040113 |
| MYLK | Myosin Light Chain Kinase | NM_053025 |
| NCF2 | Neutrophil cytosol factor 2 | NM_000433 |
| NCF4 | Neutrophil cytosol factor 4 | NM_000631 |
| NF1 | Neurofibromin 1 | NM_000267 |
| NLRC4 | NLR Family CARD Domain Containing 4 | NM_021209 |
| NLRP1 | NLR Family Pyrin Domain Containing 1 | NM_033004 |
| NLRP12 | NLR Family Pyrin Domain Containing 12 | NM_144687 |
| NLRP3 | NLR Family Pyrin Domain Containing 3 | NM_001243133 |
| NLRP6 | NLR Family Pyrin Domain Containing 6 | NM_138329 |
| NLRP7 | NLR Family Pyrin Domain Containing 7 | NM_001127255 |
| NOD2 | Nucleotide-binding oligomerization domain 2 | NM_022162 |
| NOTCH1 | Notch 1 | NM_017617 |
| NOTCH3 | Notch 3 | NM_000435 |
| NRAS | Neuroblastoma ras | NM_002524 |
| OSMR | Oncostatin M Receptor | NM_001323505  NM_001323506  NM_003999 |
| OTULIN | OUT deubiquitinase with linear linkage specificity | NM_138348 |
| PIK3CD | Phosphatidylinositol-4,5-bisphosphate 3-kinase, catalytic subunit delta | NM_005026 |
| PIK3R1 | Phosphatidylinositol 3-kinase regulatory subunit alpha | NM_181504 |
| PLCG2 | Phospholipase C, Gamma-2 | NM_002661 |
| PLOD1 | Procollagen-lysine, 2-oxoglutarate 5-dioxygenase 1 | NM_001316320 |
| POMP | Proteasome maturation protein | NM_015932 |
| PRF1 | Perforin | NM_005041 |
| PRG4 | Proteoglycan 4 | NM_005807 |
| PRKCD | Protein Kinase C, Delta | NM_006254 |
| PRKG1 | Protein kinase, cGMP-dependent, type I | NM_001098512 |
| PSMA3 | Proteasome Subunit Alpha 3 | NM_002788 |
| PSMB4 | Proteasome Subunit Beta 4 | NM_002796 |
| PSMB8 | Proteasome Subunit Beta 8 | NM_148919 |
| PSMB9 | Proteasome Subunit Beta 9 | NM_002800 |
| PSTPIP1 | Proline-Serine-Threonine Phosphatase Interacting Protein 1 | NM_003978 |
| PTEN | Phosphatase and tensin homolog | NM_000314 NM_001304717 |
| PYCARD | PYD and CARD domain containing | NM_013258 |
| RAB27A | RAB27A, Member RAS Oncogene Family | NM_004580 |
| RAG1 | Recombinant activating gene 1 | NM_000448 |
| RANBP2 | RAN Binding Protein 2 | NM_006267 |
| RASGRP1 | RAS Guanyl Releasing Protein 1 | NM_001128602  NM_005739 |
| RBCK1 | RANBP2-Type And C3HC4-Type Zinc Finger Containing 1 | NM_031229 |
| RET | Ret Proto-Oncogene | NM_020975 |
| RHOD | Ras Homolog Family Member D | NM_014578 |
| RNASEH2A | Ribonuclease H2 subunit A | NM_006397 |
| RNASEH2B | Ribonuclease H2 subunit B | NM_024570 |
| RNASEH2C | Ribonuclease H2 subunit C | NM_032193 |
| RNF213 | Ring Finger Protein 213 | NM_001256071 |
| SAMHD1 | SAM-domain and HD-containing protein 1 | NM_015474 |
| SCN9A | Sodium channel, voltage-gated, type IX, alpha subunit | NM_002977 |
| SERPING1 | Serpin Peptidase Inhibitor, Clade G (C1 Inhibitor), Member 1 | NM_000062 |
| SH2D1A | SH2-domain protein 1a (Slam-associated protein) | NM_002351 |
| SH3BP2 | SH3-domain binding protein 2 | NM_003023 |
| SKI | v-ski avian sarcoma viral oncogene homolog | NM_003036 |
| SKIV2L | Superkiller viralicidic activity 2-like | NM_006929 |
| SLC29A3 | Solute carrier family 29 (nucleoside transporter), member 3 | NM_018344 |
| SLC2A10 | Solute carrier family 2 (facilitated glucose transporter), member 10 | NM_030777 |
| SLC37A4 | Solute carrier family 37 (glucose-6-phosphate transporter), member 4 | NM_001467 |
| SLC7A7 | Solute Carrier Family 7 Member 7 | NM_001126106 |
| SMAD2 | SMAD Family Member 2 | NM_005901 |
| SMAD3 | SMAD family member 3 | NM_005902 |
| SMAD4 | SMAD family member 4 | NM_005359 |
| STK4 | Serine/Threonine Kinase 4 | NM_006282 |
| STX11 | Syntaxin 11 | NM_003764 |
| STXBP2 | Syntaxin binding protein 2 | NM_006949 |
| TGFB2 | transforming growth factor, beta 2 | NM_001135599 |
| TGFB3 | Transforming growth factor, beta-3 | NM_003239 |
| TGFBI | Transforming Growth Factor Beta Induced | NM_000358 |
| TGFBR1 | Transforming growth factor-beta receptor, type 1 | NM_004612 |
| TGFBR2 | Transforming growth factor-beta receptor, type 2 | NM_001024847 |
| TMEM107 | Small Nucleolar RNA, C/D Box 118 | NR_033294 |
| TMEM173 | Transmembrane protein 173 | NM_198282 |
| TNFAIP3 | TNF Alpha Induced Protein 3 | NM_001270507 |
| TNFRSF11A | TNF Receptor Superfamily Member 11a | NM_003839 |
| TNFRSF1A | TNF Receptor Superfamily Member 1A | NM_001065 |
| TRAP1 | TNF Receptor Associated Protein 1 | NM_016292 |
| TREX1 | Three prime repair exonuclease 1 | NM_016381 |
| TRIM28 | Tripartite Motif Containing 28 | NM_005762 |
| TRNT1 | tRNA nucleotidyl transferase, CCA-adding, 1 | NM_182916 |
| TTC37 | Tetratricopeptide repeat domain 37 | NM_014639 |
| TTR | Transthyretin | NM_000371 |
| UNC13D | Unc-13 Homolog D | NM_199242 |
| USB1 | U6 SnRNA Biogenesis Phosphodiesterase 1 | NM_024598 |
| USP18 | Ubiquitin-specific protease 18 | NM_017414 |
| VPS13B | Vacuolar protein sorting 13 homolog B (yeast) | NM_017890 |
| WAS | Wiskott-Aldrich syndrome | NM_000377 |
| WDR1 | WD Repeat containing 1/Actin-interacting protein 1 | NM_005112 |
| XIAP | X-linked inhibitor of apoptosis | NM_001167 |
| YY1AP1 | YY1 Associated Protein 1 | NM_001198906  NM_139118 |
